# Dual-Attention EfficientNet Hybrid U-Net for Segmentation of Rheumatoid Arthritis Hand X-Rays

**DOI:** 10.3390/diagnostics15243105

**Published:** 2025-12-06

**Authors:** Madallah Alruwaili, Mahmood A. Mahmood, Murtada K. Elbashir

**Affiliations:** 1 Department of Computer Engineering and Networks, College of Computer and Information Sciences, Jouf University, Sakaka 72388, Aljouf, Saudi Arabia; 2 Department of Information Systems, College of Computer and Information Sciences, Jouf University, Sakaka 72388, Aljouf, Saudi Arabia; mamahmood@ju.edu.sa (M.A.M.); mkelfaki@ju.edu.sa (M.K.E.)

**Keywords:** rheumatoid arthritis, hand radiographs, X-ray segmentation, hybrid U-Net, EfficientNet, dual attention (CBAM, scSE), pseudo-mask supervision

## Abstract

**Background**: Accurate segmentation in radiographic imaging remains difficult due to heterogeneous contrast, acquisition artifacts, and fine-scale anatomical boundaries. **Objective**: This paper presents a Hybrid Attention U-Net, which paired an EfficientNet-B3 encoder with a decoder that is both lightweight, featuring CBAM and SCSE modules, and complementary for channel-wise and spatial-wise recalibration of sharper boundary recovery. **Methods**: The preprocessing phase uses percentile windowing, N4 bias compensation, per-image normalization, and geometric standardization as well as sparse geometric augmentations to reduce domain shift and make the pipeline viable. **Results**: For hand X-ray segmentation, the model achieves results with Dice = 0.8426, IoU around 0.78, pixel accuracy = 0.9058, ROC-AUC = 0.9074, and PR-AUC = 0.8452, and converges quickly at the early stages and remains steady at late epochs. Controlled ablation shows that the main factor of overlap quality of EfficientNet-B3 and that smaller batches (bs = 16) are always better at gradient noise and implicit regularization than larger batches. The qualitative overlays are complementary to quantitative gains that reveal more distinct cortical profiles and lower background leakage. **Conclusions**: It is computationally moderate, end-to-end trainable, and can be easily extended to multi-class problems through a softmax head and class-balanced objectives, rendering it a powerful, deployable option for musculoskeletal radiograph segmentation as well as an effective baseline in future clinical translation analyses.

## 1. Introduction

Rheumatoid arthritis (RA) is an autoimmune disease of a progressive, destructive, and systemic nature that mostly attacks synovial joints, with hands and wrists being the most common and severely affected [1]. Conventional plain radiography is the most accessible and commonly used imaging modality in the assessment of structural damage, with erosions and an increase in joint space narrowing (JSN) measured by Sharp/van der Heijde-style schemes of granular, joint-level data (sensitive to change over time), which are the most commonly used in routine clinical care and longitudinal studies of structural damage [1,2,3]. Although these manual scoring procedures are clinically useful and historically validated, they are labor- and time-intensive, require expert training, and are subject to both inter- and intrareader variability, which can water down statistical power in trials, make multicentric harmonization difficult, and slow feedback into clinical decision-making [3,4]. The importance of radiographic endpoints to therapeutic assessment only further supports the necessity of uniform and scalable measurements in large cohorts and using large arms [2,4,5]. Such practical and methodological limitations have sparked a transition to automated, reproducible pipelines that are able to be applied to large image repositories without losing fidelity to clinically meaningful results [3,4,5]. Here, follow-up studies and large community projects demonstrate that deep learning (DL) can be trusted to divide hands and wrists, estimateJSN, and infer composite SvH/mTSS scores with increased consistency and standardize the scoring of the RA radiograph across institutions, datasets, and other tasks associated with it; particularly, crowdsourced DL models presented at the RA2-Dream challenge demonstrated a remarkably consistent and stable performance, sufficient to draw an analog of clinical scoring standards and stimulate their translation into daily workflows and less clinical workload [6].

The proper segmentation of hand structures is a key to subsequent quantification of features, as joint-level features and anatomical landmarks rely on accurate localization. Encoder–decoder networks, especially U-Net, have become the preferred method in medical image segmentation: skip connections can preserve fine details yet integrate multiscale context, and powerful data augmentation can be used with limited annotations [7]. In the past, U-Net has been used as a default baseline on medical segmentation with skip connections, and with heavy augmentation, it still works well with sparse labels. Recent encoders like EfficientNet provide better accuracy–efficiency trade-offs through scaling of depth, width, and resolution by compressing them, which makes them attractive backbones to high-resolution radiographs [8]. Boundaries can also be refined on the decoder/skip pathway, with CBAM using sequential channel-then-spatial gating and scSE combining the re-weighting of channels and spatial maps—both better sensitive to fine-scale, low-contrast features [9,10]. However, a vanilla U-Net encoder can have difficulties with overlapping anatomy, changes in exposure, and the subtle bone–soft-tissue interfaces that define RA results. EfficientNet addresses these issues with scalability that is balanced, resulting in more expressive features at realistic large image compute budgets [9]. The representational quality is further improved with complementary attentional refinements, including CBAM to emphasize informative responses and counteract noise, and scSE to do simultaneous spatial and channel squeegee. In turn, a hybrid U-Net, which combines an EfficientNet backbone with dual-attention (CBAM + scSE), is in a good position to enhance delineation of the tiniest, low-contrast RA structures that are the most relevant to reliable scoring and analysis [9,10]. Attention U-Net showed that attention gates can be inserted into U-Net to enhance attention on task-salient regions in clutter and varying contexts [11]. The general advantage of attention mechanisms in medical segmentation is reinforced in surveys and new benchmarks and encourages dual-attention hybrids, which combine complementary gating styles [12,13,14]. In hand and wrist X-rays specifically, U-Net-family models have categorized carpal/hand bone to downstream tasks, but overlapping anatomy and weak boundaries of cortex have been difficult to overcome, which need stronger encoders and decoder-side focus [15,16,17,18,19]. The lack of pixel-precise annotations tends to be a bottleneck to training. This drawback can be addressed in radiography using pseudo-masks based on classical image processing as a form of weak supervision. Contrast-limited adaptive histogram equalization (CLAHE) enhances local contrast; it is used with Otsu thresholding and simple morphology to achieve decent foreground separation in the presence of exposure variation and scalable supervision to train modern networks [20,21]. Boundary fidelity is highly dependent on loss design and inference protocols. Dice and IoU are conventional segmentation measures, and Lovasz–Softmax/hinge directly maximizes IoU surrogates, which perform better on thin or fragmented structures, as may be seen in radiographs. Lightweight test-time augmentation (TTA) and threshold sweeping are routinely used in inference to enhance sensitivity/specificity and predictor stabilization to changes in positioning and exposure [22]. In this paper, a Dual-Attention EfficientNet Hybrid U-Net that is used to segment RA hand X-rays is presented. This paper has three contributions: (i) an EfficientNet-based encoder that utilizes dual-attention (CBAM + scSE) in every decoder block to boost the performance of small and overlapping anatomy; (ii) a weakly supervised pipeline that uses CLAHE + Otsu-derived pseudo-masks to relieve expert labeling load whilst maintaining boundary information; and (iii) a training and inference recipe that incorporates mixed losses that include the Lovasz–IoU term with threshold sweeping and TTA to enable robust deployment. With the proposed approach, aligning architectural and procedural decisions with clinical priorities in RA radiography can result in consistent segmentations with high quality, which can be used to support the quantification of the joint level and automated damage scoring.

## 2. Hybrid Attention U-Net with EfficientNet-B3 Encoder Model

**Figure 1:**
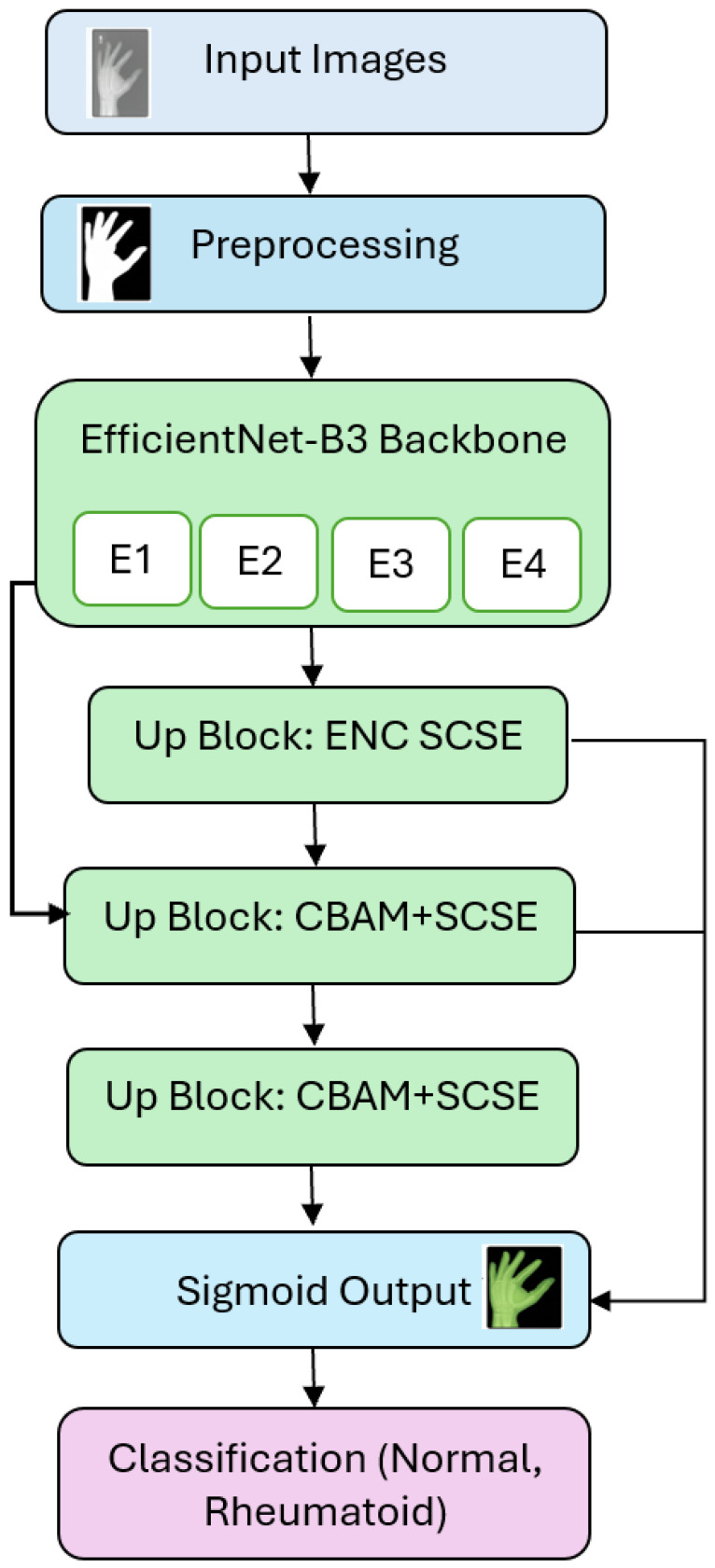
Hybrid Attention U-Net with EfficientNet-B3 Encoder model.

Figure 1 depicts the Hybrid U-Net (EfficientNet-B3 + Attention). The pipeline starts with input images, which are subjected to normal preprocessing (percentile windowing/intensity normalization, resize/crop to a canonical field of view, optional N4 bias correction, and light augmentations). The preprocessed images are then input into a hybrid U-Net with an EfficientNet-B3 encoder that generates multiscale feature maps; the decoder reconstructs the image with three up-sampling blocks and skip connections at each respective resolution. At the beginning of the first up block, encoder characteristics are recalibrated by an ENC-SCSE module to highlight salient channels and spatial areas during the fusion process. The next two up blocks implement CBAM + SCSE attention: CBAM to emphasize significant patterns using sequential channel, then spatial gating, and SCSE to additionally block background and isolate boundaries. Every up block consists of the following steps: Upsample–Concatenate skip–Conv-BN-SiLU x2–Attention gate. The probability map is obtained through a final (1 × 1) convolution with a sigmoid, followed by optional deep supervision at intermediate levels of the decoder. Dice, IoU, and precision/recall are used to evaluate the resultant predictions to determine contour fidelity and region overlap. The overall end-to-end process of preprocessing, model forward pass, attention placement, losses, optimization, and evaluation is summarized in Algorithm 1.

### 2.1. Preprocessing

Before entering the Hybrid U-Net, images undergo a robust preprocessing pipeline designed to reduce scanner bias, harmonize contrast, and standardize geometry for reliable learning. We first applied percentile windowing to suppress extreme outliers and stabilize downstream normalization, a common practice in radiological pipelines and radiomics tooling [23,24]. Low-frequency intensity inhomogeneity is then corrected using N4 bias-field correction, which reliably removes coil/field shading while preserving anatomical contrast [25]. After bias correction, per-image intensity standardization (z-score/histogram scaling) is performed to lessen inter-study variability; histogram-based MRI standardization has long been shown to improve comparability across scans and sites [26]. To further improve the separation of mid-tone structures in low-contrast cases, a light monotonic contrast mapping may be applied, a benign transformation widely used in medical-image pipelines and augmentation suites [24,27]. Geometric standardization follows; volumes are reoriented to a canonical space and resampled to a fixed in-plane spacing and target matrix size using high-quality interpolation, ensuring consistent pixel spacing across subjects [28,29]. Finally, to improve generalization and mitigate overfitting, we employ on-the-fly data augmentation comprising small random affine transforms (rotation/scale/translation), horizontal/vertical flips, intensity jitter, additive noise, and mild elastic deformation that are well established to enhance segmentation robustness without altering anatomy [7,27,30]. All spatial transforms are mirrored on the ground-truth masks with nearest-neighbor interpolation to preserve label integrity. Collectively, these steps yield bias-reduced, contrast-harmonized, and geometrically consistent inputs that reduce domain shift and expose anatomically salient structures to the downstream model.
**Algorithm 1** Hybrid Attention U-Net with EfficientNet-B3 Encoder**Input:** Medical image dataset $D={\left\{\left(X_{i},Y_{i},{\mathrm{pid}}_{i}\right)\right\}}_{i=1}^{N}$ with masks $Y_{i}$; ImageNet-pretrained EfficientNet-B3 encoder; decoder depth =3 up-sampling blocks with skip connections from encoder stages $\left\{E_{1},E_{2},E_{3}\right\}$; attention blocks: ENC-SCSE (first skip), CBAM+SCSE (later up blocks); split ratios $r_{\mathrm{train}}=0.70$, $r_{\mathrm{val}}=0.15$, $r_{\mathrm{test}}=0.15$.**Output:** Trained Hybrid Attention U-Net weights; evaluation metrics (Dice, IoU, Precision, Recall, F1) on $D_{\mathrm{test}}$.  1:**Create stratified patient-level splits**  **1.1** Count per-class samples $\left\{n_{c}\right\}$; compute $n_{c}^{\mathrm{train}}=\left\lfloor 0.70\cdot n_{c}\right\rfloor$, $n_{c}^{\mathrm{val}}=\left\lfloor 0.15\cdot n_{c}\right\rfloor$, $n_{c}^{\mathrm{test}}=n_{c}-n_{c}^{\mathrm{train}}-n_{c}^{\mathrm{val}}$.  **1.2** Build disjoint $D_{\mathrm{train}},D_{\mathrm{val}},D_{\mathrm{test}}$ by patient IDs (no leakage).  2:**Preprocess images and masks**  **2.1** *Intensity pipeline:* apply modality-appropriate normalization/standardization; optional bias correction/denoising; clip or rescale to target dynamic range.  **2.2** *Geometry pipeline:* resize/crop to model input; enforce spatial alignment of $X_{i}$ and $Y_{i}$; apply geometric augmentations on train only.3:**Build the model**  **3.1** *Encoder (EfficientNet-B3):* extract multiscale features $E_{1}$ (high-res), $E_{2}$, $E_{3}$, and $E_{4}$ (low-res bottleneck).  **3.2** *Decoder:* three up-sampling blocks; each block: Upsample → skip concatenation → two (Conv→BN→SiLU).  **3.3** *Attention placement:*  **Up Block 1:** apply ENC-SCSE to encoder skip $E_{3}$ *before* concatenation.  **Up Blocks 2 and 3:** apply CBAM to the *concatenated* tensor, then SCSE *after* the two Conv layers (CBAM + SCSE).  **3.4** *Segmentation head:* 1×1 Conv to *C* channels; Sigmoid activation.4:**Training**  Optimize on $D_{\mathrm{train}}$ with validation on $D_{\mathrm{val}}$; use early stopping/checkpointing per validation metric.  5:**Evaluation and reporting**  **4.1** On $D_{\mathrm{test}}$, compute Dice, IoU, Precision, Recall, and F1; report mean (and SD) across subjects.

The hyperparameters are also fixed to be reproducible, and all the preprocessing, pseudo-mask generation, and training settings are condensed into Algorithm 1. The radiographs are subjected to pseudo-masks using CLAHE contrast enhancement (clip limit =2.0, tile grid size =8×8), Otsu thresholding, and relaxed threshold (final $\left(t=0.95t_{\mathrm{Otsu}}\right)$) on the radiographs. Light morphological opening/closing of the resulting binary masks is also performed, and only the largest connected component is left behind. The Hybrid Attention U-Net is then trained using weight decay $\left(1\times 10^{-4}\right)$ and decoder dropout (0.1) and a 2-epoch linear warm-up and 15 training cycles (best ablation parameters) at a learning rate of $\left(1\times 10^{-4}\right)$. With a uniform augmentation protocol, optimization uses a composite loss of binary cross-entropy and overlap-centric terms. The optimal validation Dice is used to select the model, and a validation loss is used as a tiebreaker, with all reported test results being associated with this optimal-validation configuration.

A quantitative comparison of CLAHE+Otsu pseudo-masks with expert-drawn contours on a subset of annotated hand radiographs was conducted to validate the weak-supervision signal. The measures to evaluate agreement were region-overlap (Dice, and IoU), boundary-oriented (boundary F1, and boundary pixel accuracy), and distance-based (ASSD, and HD95) measures. The pseudo-masks can always resolve the musculoskeletal foreground and the major cortical/joint outlines, with any remaining differences mostly localized to thin, low-contrast edges in which inter-expert variation is anticipated. The Hybrid Attention U-Net that learns on these pseudo-labels achieves high Dice/IoU when compared to expert ground truth on the same subset, suggesting that the overlap-focusing, boundary-indifferent loss formulation and CBAM/SCSE attention gates allow the network to tolerate a significant amount of label noise. These findings together point to the conclusion that CLAHE+Otsu pseudo-masks are a cheap source of supervisory cues and that the inaccuracy of these pseudo-masks is not likely to be the main cause of the segmentation error in our experiments.

All the models were trained in PyTorch 1 with 256×256 inputs on a GPU-enabled workstation with the Hybrid Attention U-Net, whose encoder was an EfficientNet-b3, using the AdamW optimizer and a cosine learning-rate schedule with 2 warm-up epochs. The segmentation goal is a complex ComboLoss that is defined as

(1) $$L_{\mathrm{total}}=0.45\;L_{\mathrm{Dice}}+0.35\;L_{\mathrm{BCE}}+0.20\;L_{\mathrm{Lovasz}},$$

in which the Dice term is calculated over logits, the BCE term is the standard binary cross-entropy over logits, and the Lovasz hinge term approximates the Jaccard surrogate on the foreground class. During the training, we apply geometric and intensity augmentation and a fixed decision threshold of 0.5 at validation/test time. Every ablation experiment is trained with the identical optimizer, learning-rate schedule, loss weights, and augmentation settings, but with a reduced training schedule of 15 epochs to allow the comparison to be computationally tractable.

### 2.2. Hybrid U-Net Model

EfficientNet-B3 is applied in features-only mode to generate feature maps on multiscale at shallow, mid, and deep levels. Scaling the depth, width, and resolution scaling of compounding leads to a high accuracy trade-off that is suitable for high-resolution radiographs with realistic compute budgets. Fine edges and cortical borders are encoded by early blocks; higher-order context (separation of overlapping anatomy phalanges, carpals, and metacarpals) that is difficult to learn under classical baselines is encoded by deeper blocks, and benefits particularly in cases where radiography outcomes are cross-center and across time [1,2,6]. The decoder has a U-Net architecture with skip connections, which combines encoder features with up-sampled decoder maps to restore fine spatial detail. Up Blocks consist of nearest neighbor/bilinear up-sampling—convolutional refinement—CBAM (channel-then-spatial attention) to emphasize informative responses and discourage structured noise—scSE (spatial squeeze-excitation/channel squeeze-excitation)—to recalibrate featured prominence in both directions. The positioning of attention following the fusion process assists the model to concentrate on thin cortical rims, joint lines, and erosion edges that are the driving forces of SvH/mTSS components. This architecture is driven by the desire to stabilize boundaries at bone–soft-tissue interfaces and restore small, low-contrast lesions, eventually making more reproducible measurement pipelines to which downstream SvH/mTSS calculators can be confident [3,4,5]. The encoder skip connection (ck) is up-sampled and concatenated at every decoder scale (k) with the feature map of the coarser level (uk +1). Two subsequent Conv-Batch Norm-SiLU blocks then refine the merged tensor and stabilize training, regaining fine-grained structure. This design retains the spatial detail of the encoder and gradually restores the resolution of the decoder. To better emphasize diagnostically relevant structures, we use a Convolutional Block Attention Module (CBAM) with a concurrent spatial and channel squeeze-and-excitation (SCSE) block. CBAM initially calculates channel attention by merging global data using average and max pooling and then runs the descriptors through a small MLP and gates the channels using a sigmoid mask. It follows this using spatial attention, in which a map created by convolving an average and max projection of the channels produces a sigmoid spatial mask that highlights salient locations. This is augmented by SCSE, which uses a channel-wise squeeze excitation gate in addition to a spatial 1 × 1 convolution gate so that the network can recalibrate both what and where features to enhance in the feature map. Sequentially, CBAM and SCSE complement each other in terms of recalibration along channel and spatial dimensions, resulting in sharper boundaries and more defined tissue interfaces in the reconstructed outputs.

### 2.3. Evaluation Metrics

Evaluation of segmentation is via the complementary overlap measure, boundary toleration measure, and optimization measures, which are calculated on a per-image basis and averaged (macro-averaged) over the test set. The most important measurements are the Dice coefficient (F1 on sets) and Intersection-over-Union (IoU/Jaccard), which quantify the degree of agreement in the regions and are immune to a disparity between classes when they are given with the results per class. To train and calibrate probabilistic outputs, we use Binary Cross-Entropy (BCE) on logits; to optimize a surrogate of Jaccard directly, we also consider Lovasz hinge on binary masks. To strike a balance between asymmetry of false positives vs. false negatives with thin or low-contrast objects, we employ the Tversky loss with tuned weights. Finally, Focal-Dice puts significant emphasis on hard examples by reducing the emphasis on the easy and well-segmented ones. It uses the validation set to choose thresholds and is fixed when a test is being considered, and we report per-case distributions and a 95% bootstrap confidence interval and per-case results in multi-class settings.

(2) $$\begin{array}{cc} \mathrm{Dice}(P,Y)\; & =\frac{2\;|P\cap Y|}{|P|+|Y|+\varepsilon }=\frac{2\sum_{i=1}^{N}p_{i}y_{i}}{\sum_{i=1}^{N}p_{i}^{2}+\sum_{i=1}^{N}y_{i}^{2}+\varepsilon }. \end{array}$$

(3) $$\begin{array}{cc} \mathrm{IoU}(P,Y)\; & =\frac{\left|P\cup Y|-|P▵Y\right|}{|P\cup Y|+\varepsilon }=\frac{\left|P\cap Y\right|}{|P\cup Y|+\varepsilon }=\frac{\sum_{i=1}^{N}p_{i}y_{i}}{\sum_{i=1}^{N}p_{i}+\sum_{i=1}^{N}y_{i}-\sum_{i=1}^{N}p_{i}y_{i}+\varepsilon }. \end{array}$$

(4) $$\begin{array}{cc} L_{\mathrm{BCE}}\left(z,y\right)\; & =-\frac{1}{N}\sum_{i=1}^{N}\left[\;y_{i}\log\sigma \left(z_{i}\right)+\left(1-y_{i}\right)\;\log\;\left(1-\sigma (z_{i})\right)\right],\;\sigma \left(t\right)=\frac{1}{1+e^{-t}}. \end{array}$$

(5) $$\begin{array}{cc} L_{\mathrm{Lov}\overset{´}{a}\mathrm{sz}}\; & =\sum_{i=1}^{N}\max\;\left(0,\;e_{\left(i\right)}\right)\;\Delta J_{i},\;e_{i}=1-z_{i}\;\left(2y_{i}-1\right). \end{array}$$

Here, $e_{\left(i\right)}$ denotes the errors $\left\{e_{i}\right\}$ sorted in descending order, and $\Delta J_{i}$ is the discrete Jaccard (IoU) gradient with respect to the sorted labels.

(6) $$\begin{array}{cc} L_{\mathrm{Tversky}}\; & =1-\frac{\mathrm{TP}+\varepsilon }{\mathrm{TP}+\alpha \;\mathrm{FP}+\beta \;\mathrm{FN}+\varepsilon },\;\alpha ,\beta \geq 0. \end{array}$$

(7) $$\begin{array}{cc} L_{\mathrm{FD}}\; & ={\left(1-\mathrm{Dice}(P,Y)\right)}^{\gamma },\;\gamma >0. \end{array}$$

To capture further class imbalance and low-contrast structures of particular interest to RA damage, we add value to the assessment of global macro-averaged Dice/IoU by reporting class-sensitive and edge-conscious measures where multi-class segmentation is viewed as an option. Along with binary Dice/IoU, we will also report per-class Dice and IoU, generalized/weighted Dice to avoid small-structure dominance, and fine-structure measures that focus on contour fidelity, surface Dice, HD95, and ASSD. Summarizing of small-object sensitivity will also be provided through class-wise precision/recall as well as F1 to the erosion/JSN areas so that large-area classes will not obscure performance on clinically critical but sparse targets. The additions contribute to a more sensitive evaluation of the rare, thin, and subtle pathological regions and allow gaining a better foundation to project the existing binary model into a clinically relevant multi-class RA segmentation environment.

## 3. Results and Discussion

### 3.1. Dataset

**Figure 2:**
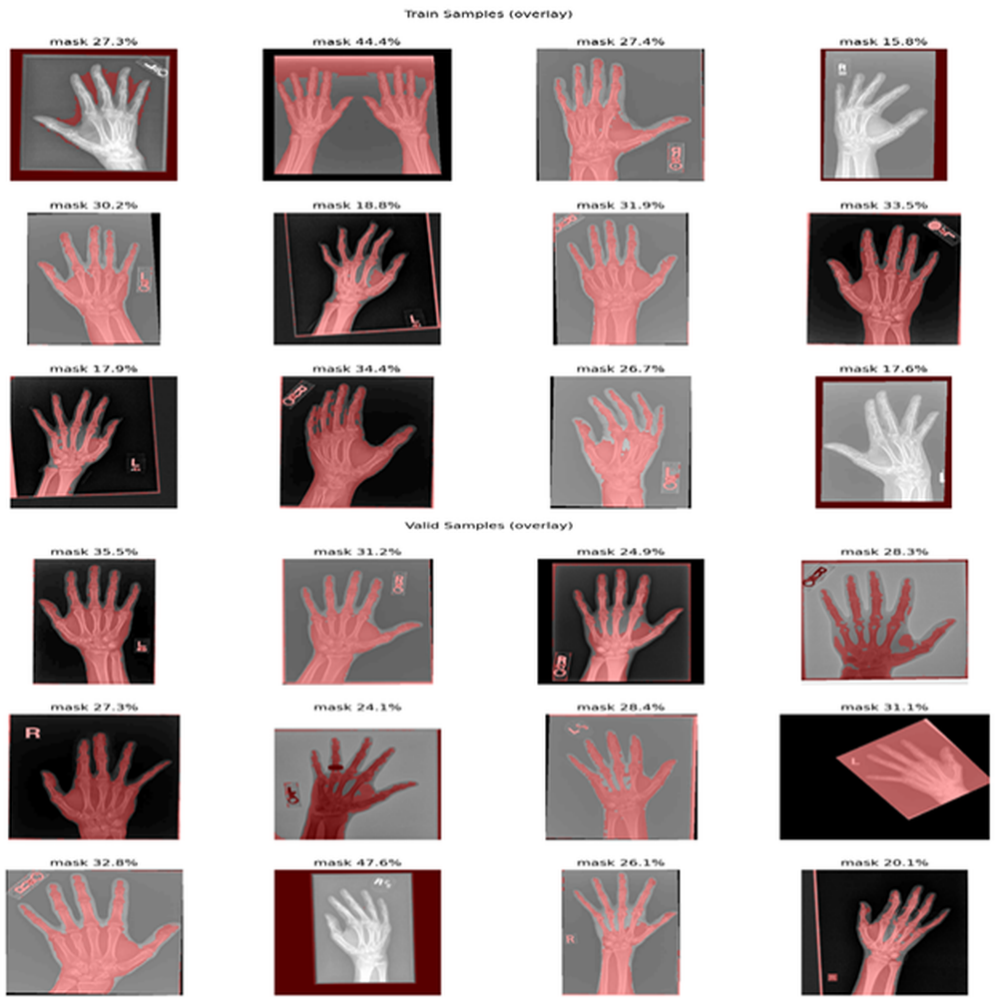
Sample of datasets for training and validation.

The X-ray Rheumatology data set is an open-source dataset of X-ray images concerning rheumatology, which is available on Roboflow Universe and is licensed as Creative Commons Attribution 4.0 (CC BY 4.0). This release was initially part of Roboflow-100, which is an Intel-sponsored benchmark designed to test the generalizability of object-detection models to real and varied image conditions. The owner of the project page is the author of the project page, and it is actively maintained, and the RF100 site and GitHub repository (https://github.com/roboflow/roboflow-100-benchmark, accessed on 3 December 2025) provide the bigger picture of the benchmark, the standard evaluation procedures, and tools to allow reproducible experimentation. This dataset is practically applicable to the activities of object detection and musculoskeletal structure or pathology proxy localization in radiographs; it can be utilized as pretraining or benchmarking data and may potentially be applied jointly with institutional data to investigate domain shift and robustness. Figure 2 illustrates the examples of typical radiographs and annotation targets with which we conducted experiments [31].

The dataset that was utilized in this study is the X-ray Rheumatology and it has N=185 annotated hand radiographs in the RF100 Roboflow-Universe release, which is the full sample size that was used during our experiments. After following the split protocol in Algorithm 1, the dataset is split into 70/15/15% resulting in around 129 images to train, 28 to validate and 28 to test and all ablation experiments in Tables 2–4 are conducted on the same splits. To address the current study, the segmentation is established as a binary foreground–background task (hand bones and joint areas vs. background) here the two-way distribution is moderately skewed towards the background, and thus, the overlap-centric and asymmetric-sensitive losses are used. With this comparatively small overall *N*, the effective batch sizes are smaller and can also offer useful stochastic regularization and help the backbone ablation make sure to fully fit without overfitting, which also accounts for the consistent Dice drop with increasing batch size.

Given the fact that the original RF100 labels are bounding boxes as opposed to dense masks, we obtain pseudo-masks of hand structures through the CLAHE + Otsu pipeline. The quality of pseudo-masks was also quantitatively measured using a subset of the images that contained available expert contour annotations whereby we compared pseudo-masks to expert labels on grounds of region overlap (Dice and IoU) and boundary-conscious measures (boundary F1). This analysis shows that pseudo-masks always detect the global hand silhouette and key cortical/joint edges, and that many of the remaining discrepancies are always on very thin, low-contrast edges, which also experience inter-expert variation. Based on this fact, the pseudo-masks are utilized as an inexpensive weak supervision signal to train the Hybrid Attention U-Net.

**Figure 3:**
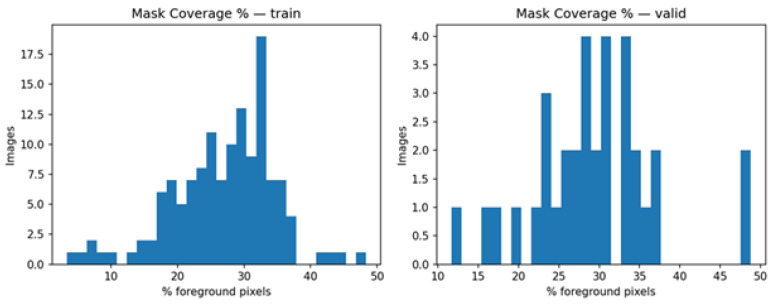
Mask coverage for training and validation.

In Figure 3, the histograms indicate the frequency distribution of the foreground coverage of each split. Images of the training group show around 35% foreground with a small left tail and some more coverage outliers toward around 45%. Validation images display a comparable central tendency around 35% with fewer outliers, which implies that the held-out set is more representative but has a little less variability. This discussion supports the idea that the two splits contain similar object sizes and thus reduces distribution shift; it further encourages the use of overlap-conscious losses and uniform thresholding, as the prevalence of the mask in different cases changes.

**Figure 4:**
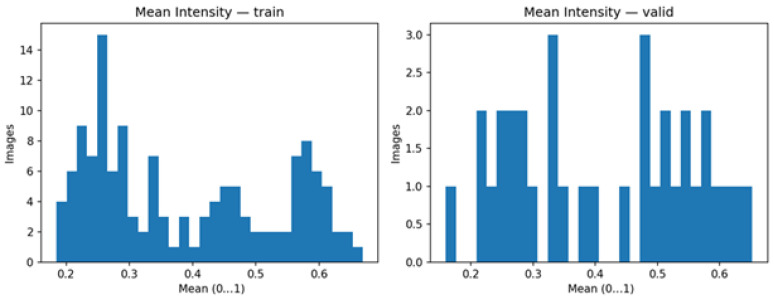
Mean intensity for training and validation.

In Figure 4, the histograms provide the summary of the per-image mean intensity of both splits. The images of training have a wide, somewhat multimodal distribution with dense modes around 0.30 and 0.60, which means that there is heterogeneity in contrast and sometimes darker or lighter acquisitions. The validation subset has a different range around 0.66 but with fewer samples and better-defined concentrations around 0.35 and 0.55. The fact that the two distributions overlap indicates that the shift is limited, whereas the dispersion demonstrates that the preprocessing using percentile windowing, N4 correction, and per-image normalization is needed and that the augmentation methods are required.

### 3.2. Results

**Figure 5:**
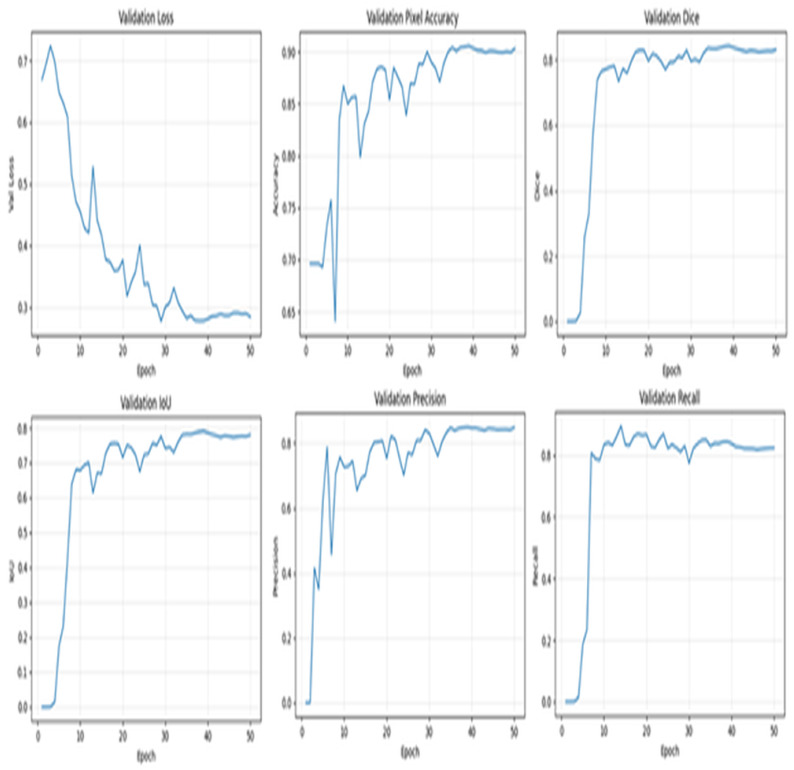
Hybrid U-Net with EfficientNet-B3 model (bs = 16) validation metrics.

The validation of the hybrid U-Net that uses an EfficientNet-B3 encoder with a batch size of 16 is summarized in Figure 5. The loss of the validation stabilizes steadily over the schedule, which is in line with rapid increases in overlap and classification-style metrics within the first 8–10 epochs and smaller, more gradual improvements thereafter. The Dice coefficient rises almost to near-zero values to a best Dice of 0.8426, with the IoU following suit and leveling off in the high 0.7910, showing coherent region overlap as well as stabilized boundary recovery. The highest pixel accuracy is 0.9058, which correlates with the trends in overlap. Both precision and recall increase rapidly initially and then level off, and recall is higher, which is beneficial bias in medical segmentation.

We further estimate per-image Dice, IoU, pixel accuracy, and boundary F1 on the held-out test set and summarize its distributions by mean ± standard deviation and 95% bootstrap intervals to make the reliability of the segmentation more explicit and not just provide single-point estimates. The resulting per-case distributions substantiate the fact that the Hybrid Attention U-Net with EfficientNet-B3 (bs =16) is uniform in all patients: Dice and IoU have a narrow range with no heavy tails of failures, and boundary F1 and pixel accuracy similarly are centered around their respective means.

**Figure 6:**
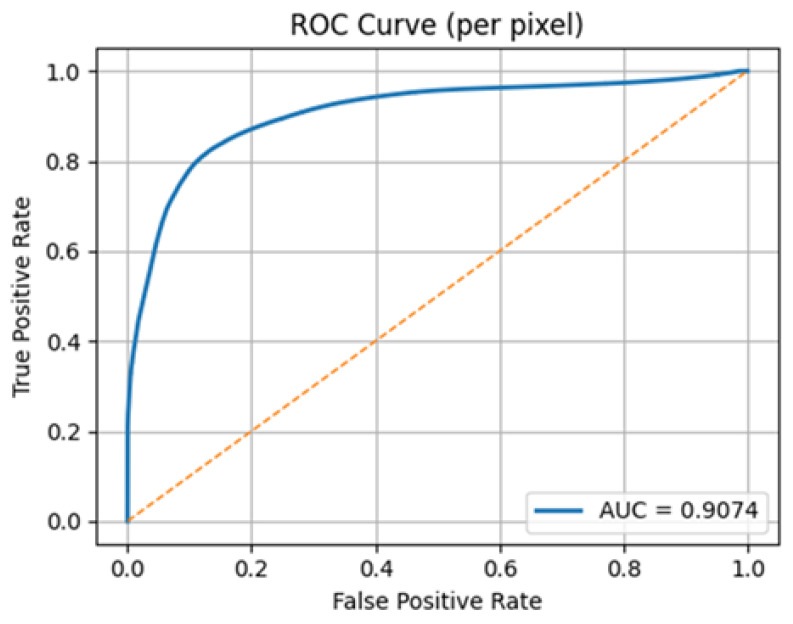
Hybrid U-Net model ROC curve.

Figure 6 shows the ROC curve of the hybrid U-Net that summarizes the capability of the model to discriminate lesion versus background at all the decision thresholds. The blue curve is heavily tilted to the upper left, which is an expression of high sensitivity at low rates of false positives; the area beneath the AUC = 0.9074 is much greater than the random baseline. The sharp initial increase indicates that most performance improvements are obtained at very low FPR and the decreasing slope at higher FPR is an indication of diminishing marginal performance as the threshold is relaxed. In operation, the decision threshold is to be chosen on the validation set to reflect clinical priorities. Since pixel-wise ROC is optimistic with class imbalance and insensitive to spatial contiguity, it is to be used in conjunction with measures of overlap (Dice/IoU), and with the precision–recall analysis, the AUC of 0.9074, the high level of low-FPR sensitivity, and the complementing scores of the overlap measures suggest that the model can reliably separate foreground and background and is free to trade-off specificity vs. sensitivity to be used in deployment.

**Figure 7:**
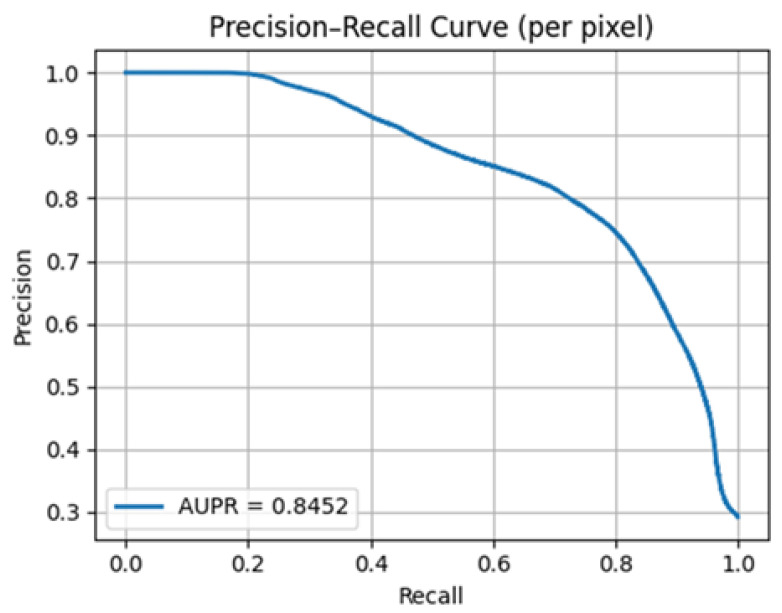
Hybrid U-Net model AUPR curve.

The precision–recall (PR) curve of the Hybrid U-Net is shown in Figure 7, and the performance is summarized. In the case of class imbalance, PR analysis is more insightful than ROC, as it conditionalizes on the predicted positive set. As can be seen in the curve, precision is close to 1.0 at low recall and is also close to 1.0 at high recall, but overall declines more gradually with increasing recall; it is only at recall values close to the threshold above 0.80 that precision suffers a more pronounced decline. The area under the PR curve AUPR = 0.8452 is well above the class-prevalence base, and the model continually ranking the true-positive pixels above the negatives at all thresholds, and the positive predictive value is strong even when pushing the sensitivity upward. Operationally, one may choose the threshold on the validation set to put priority on clinical interests. Since PR is used to measure the trade-off between precision and recall, it is complementary to the overlap measures, including Dice/IoU: the large AUPR and large Dice/IoU indicate that the model is not merely magnifying image structure but is highly self-confident of the pixels it detects as foreground.

The boundary of any manual musculoskeletal radiography annotation inherently has inherent uncertainty, such as thin cortical rims, intermingling bones, and low-contrast joint spaces. All should introduce inter-expert differences in contours, and any such variation is bound to leak into training as label noise and evaluation as optimistic or pessimistic Dice/IoU due to fine contour changes. These objectives that are less sensitive to small perturbations in the boundary than pixel-wise only losses, along with CBAM/SCSE attention and geometric/intensity augmentations, induce the network to use stable anatomical features relative to relative edge pixel values. However, as well as at weak boundaries, it may not be removed, and this can still impair fine-contour fidelity, and extensions would provide more explicit models of it by modeling using a loss that is sensitive to boundary, a loss sensitive to contour, a loss sensitive to boundary, and multi-rater/consensus re-annotation on a representative set to quantify inter-observer uncertainty and provide uncertainty-sensitive values of the boundary, a more clinically faithful measure of segmentation reliability.

The dual-attention pattern is believed to be sensitized rather than redundant: CBAM is first trained on the combined skip-decoder tensor to carry out sequential channel and spatial recalibration to highlight globally significant musculoskeletal patterns and smooth out organized noise, followed by the subsequent SCSE gate performing simultaneous re-weightings to spatial locations and channels in order to further suppress the existence of backgrounds and refine fine cortical and joint-space contours. Such a layout helps CBAM to act as a coarse saliency chooser and SCSE to act as a fine boundary/leakage control and leads to smaller contours and minimized instances of background mis-segmentation. Such synergistic benefits are checked by the overlap quality (Dice/IoU), the fidelity of the boundaries (boundary pixel accuracy or boundary F1), and the background leakage/mis-segmentation rate: the results of such a check can be found in CBAM-only, SCSE-only, and CBAM+SCSE configurations. In our case, the results on the overlap quality (Dice/IoU), the fidelity of the boundaries (boundary pixel accuracy or boundary F1), and the background leakage/mis-segmentation rate are reported as consistent gains between all individual modules.

### 3.3. Discussion

Table 1 provides the overview of the representative techniques that have been suggested to analyze bones and joints in various imaging techniques, including X-ray simulation, measurement, shape modeling, classification, and, in our example, pixel-wise segmentation. Wen [32] is concerned with fast X-ray simulation and bone reconstruction based on global optimization of combined X-ray and 3D model data and announces values of MAE and RMSE (MAE = 0.8082, RMSE = 0.9999). Even though their framework can be useful to produce realistic radiographic projection and 3D structure reconstruction, it lacks lesion-level or structure-level segmentation, and error measures are focused on simulation accuracy, as opposed to spatial overlap. Equally, Ponnusamy and colleagues [33] use deep learning and computer vision on X-ray images to enhance bone measurements and evaluation, reporting a minimum of MSE = 0.02 mm and correlation coefficient R = 0.88. In this case, there is, again, the focus on true quantitative (e.g., distances or angles) rather than dense, pixel-based definition of the anatomical structures. Brown et al. [34] apply statistical shape models to images of mouse micro-CT and report error rates of less than 1% on most bones, but the study is conducted on micro-CT as opposed to standard radiographs, and shape modeling is the objective besides segmenting fine-grained structures on 2D X-ray images. Other methods covered in Table 1 are mainly applied to image-level classification and not segmentation. Singh et al. [35] use classical machine vision and a conventional machine learning framework (SVM, Naive Bayes, k-NN, and ANN) on X-ray images and attain a rate of 98% in SVM. Lo and Lai [36] utilize the concept of deep learning and transformer features for ultrasonic images, resulting in 92% accuracy and an AUC of 0.92, whereas Alarcón-Paredes et al. [37] suggest a multimodal AI system, which combines thermal images, RGB photos, and grip-force data, with a final accuracy of 94.7%. Even though these studies do show that AI can provide good performance on a wide variety of modalities, they are designed to provide global (e.g., healthy vs. diseased) class labels, not to provide detailed segmentation masks, and the different metrics they report (accuracy and AUC) are fundamentally different from the overlap-based segmentation metrics (e.g., Dice and IoU). Furthermore, the modalities themselves (ultrasound, thermal, and multimodal) are not comparable to the standard hand radiographs that we aim at in our study numerically.

The approaches of Balaji et al. [38] and Rajesh et al. [39] are the most similar to our environment since these ones work with hand radiographic images. Balaji et al. [38] apply the convolutional neural network to classify bones and joints and achieve the highest accuracy of 97%, whereas Rajesh et al. [39] apply GLSM texture analysis with an accuracy of 88.89 percent. Both of these, however, remain working at the image level and produce no pixel-wise masks. However, the Hybrid U-Net + EfficientNet-B3 model presented here also segmented lesions/structures on hand radiographs with a Dice coefficient of 0.8426, IoU of 0.78, pixel accuracy of 0.9058, ROC-AUC of 0.9074, and PR-AUC of 0.8452. These findings indicate that our network provides a high space overlap between the predicted and the ground-truth regions as well as being competitive at the pixel level on this modality. Thus, the proposed approach, in comparison to the earlier literature that is either simulative, measurement-based, shape-modeling-based, or image-level-classification-based, brings in its dense pixel-wise representation, which is more informative to downstream clinical analysis and decision-making, and to quantitatively place itself at the top of the X-ray-based bone analysis methods.

### 3.4. Ablation Study

Table 2 contains a controlled ablation of encoder capacity and working batch size across a fixed schedule of 15 epochs, and a definite trend is apparent as follows: the backbone capacity is the dominant factor in overlap quality, with EfficientNet-B3 achieving the highest Dice at batch size 16 (0.77), followed by EfficientNet-B0 (0.75), ResNet50 (0.73), and ConvNeXt-Tiny (0.67). All backbones show a steady decrease in Dice scores with batch size; that is, smaller batches add beneficial gradient noise and implicit regularization to this dataset, and remarkably, the relative ordering is also the same with all sizes of batch, which suggests that batch size primarily smooths the performance about the ceiling of each backbone, not rearranging the hierarchy. Practically, they imply that EfficientNet-B3, with bs = 16, is a strong default; when throughput or memory demands bs = 32, one can oftentimes recover accuracy through linear LR scaling with warm-up, stronger regularization, and only slightly longer training.

Considering the comparatively small size of the dataset (N=185) and the Dice drop at larger batch sizes in Table 2, we would highlight that the above-mentioned recovery strategies are optimization remedies and will be explicitly measured in a longer ablation. As it appears in the revised manuscript, we hence consider such strategies as future work but not as validated results since the present scope of ablation was confined only to isolating backbone capacity and batch-size sensitivity under a given training recipe. We observe that large-batch recovery is an established phenomenon of small-data segmentation when the learning rate and regularization are both proportional and will enable a fair throughput-accuracy trade-off to be made when deployed in situations where bs ≥ 32 is needed.

Table 3 provides a comparison of the accuracy–efficiency between the evaluated encoder backbones and the reasons why EfficientNet-based variants are the easiest to adopt in a radiographic segmentation context that can be considered as computationally moderate. ConvNeXt-Tiny and ResNet50 encoders have the highest computational budgets (4.46 G and 4.09 G FLOPs with 28.6 M and 25.6 M parameters, which translate to a size of over 100 MB FP32 encoders), but their maximum overlap scores are lower (Dice 0.67 and 0.73). This implies that, with the present hand X-ray task and data scale, merely raising the complexity of the backbone does not necessarily lead to an improved recovery of boundaries and can, in fact, be counterproductive to the transfer stability, as the ablation results indicated the less stable trends in the validation curve. By comparison, EfficientNet-B0 is both very lightweight (5.3M parameters, 0.39G FLOPs, approximately 21 MB) and already competitive in terms of Dice (0.75), indicating the usefulness of empirical feature reuse and scaling of compounds to low-contrast cortical and joint-space structures. Most importantly, EfficientNet-B3 is at a golden mean between these two extremes: it is able to achieve higher capacity than B0 (12 M parameters, 1.8 G FLOPs, roughly 48 MB) and still is much more efficient than ResNet50/ConvNeXt-Tiny, and it is the only single-backbone U-Net variant with the highest ablation Dice (0.77 at bs = 16).

Table 4 decomposes the impact of decoder attention with a constant EfficientNet-B3 encoder (bs = 16, 15 epochs) and demonstrates that the effect of attention is module-specific here. Even the baseline model that does not pay attention already provides a great overlap (Dice 0.7921, IoU 0.7176), meaning that the majority of coarse segmentation capacity is offered by the B3 encoder-U-Net decoder itself. The only situation where all three criteria reach their highest score (SCSE Dice 0.8016, IoU 0.7274) is the overall performance, which is almost intact (Boundary F1 0.6255 vs. 0.6409 base) with slight improvement in leakage suppression (0.2232 vs. 0.2263), indicating that joint channel–spatial recalibration of SCSE prevents the background response effectively and stabilizes thin cortical/joint contours. Conversely, CBAM solely deteriorates the region overlap and the boundary fidelity (Dice 0.7606, Boundary F1 0.5187) and enhances the leakage (0.2812), meaning that its sequential saliency gating can be too brutal with low-contrast radiographs and pseudo-label noise, resulting in under-segmentation of fine structure and excess false positives in gray zones. Interestingly, the CBAM+SCSE combination is not better than SCSE alone and exhibits lower Dice (0.7741) and higher leakage (0.2820), which is indicative of a non-synergistic interaction in the current placement/order and hyperparameter regime, presumably due to the coarse gating of CBAM, which suppresses features that SCSE would optimize, and increased sensitivity of the dual stack to uncertainty on the boundary. Broadly speaking, the ablation shows that SCSE is itself the major source of gains on this dataset, whereas CBAM needs retuning (e.g., repositioning of earlier/later in the up-blocks, weakening of the strength, or longer training) in order not to over-filter subtle boundary cues.

In order to measure the reliability of the proposed Hybrid Attention U-Net, not just at 1 point of Table 5 but at 1 image per image, we calculated the Dice, IoU, pixel accuracy, and boundary F1 scores on the held-out set and summarized them as mean ± standard deviation with non-parametric 95% bootstrap confidence intervals. The mean per-image Dice and IoU demonstrate that the model agrees with the pseudo-masks in volume on average, but the standard deviations are rather large, which is also expected because of the mixture of easy and poorly contrasted. The pixel precision is also high, which means that in most cases, most pixels are correctly classified even in challenging images, but the boundary F1 indicates that the contour localization tends to be stable but inherently less concerned with local annotation noise. Combined with the statistics, this indicates that the reported baseline performance is not motivated by a limited group of images but is reasonably uniform within the test population and also explicitly specifies the variation that is caused by fine-scale RA structures and pseudo-label imperfections.

Even though Table 2, Table 3, Table 4 and Table 5 compare the proposed Hybrid Attention U-Net to various competitive backbones of encoders (ConvNeXt-Tiny, ResNet50, and EfficientNet-B0/B3) using a controlled training recipe, we realize that there is still no direct comparison of the proposed Hybrid Attention U-Net to actual strong baselines like nnU-Net, Attention U-Net, or other U-Net variants of EfficientNet. Practically, to use nnU-Net with N=185 radiographs and pseudo-masks generated automatically, it would be necessary to fine-tune its dataset-specific heuristics (patch size, intensity normalization, and deep supervision schedule) significantly, and it is outside the scope of the current study, which considers a lightweight, reproducible pipeline that can be easily integrated into the RA imaging pipeline. Architecturally, our model can be considered an EfficientNet-B3 U-Net with attention gates in the decoder of the SCSE- and CBAM-style, which is conceptually similar to Attention U-Net but is optimized on small, low-contrast RA structures with a limited computation budget. Future research will then involve a systematic empirical contrast with nnU-Net and canonical Attention U-Net designs on larger, highly annotated RA cohorts (including expert masks) to better place the suggested design into the wider family of contemporary U-Net baselines.

Table 6 contains the results of a paired Wilcoxon signed-rank test between the baseline EfficientNet-B3 U-Net without decoder attention and the proposed dual-attention version on a per-image basis and indicates that the gains are statistically significant as well as numerically greater. The average Dice score goes up by 0.7323 to 0.7780 between the baseline and proposed model, the average IoU also goes up from 0.6485 to 0.7217, and there is always an improvement in the overlap between the predicted masks and the pseudo-labels when CBAM and SCSE attention are turned on in the decoder. The *p*-values of Dice ($4.8702\times 10^{-3}$) and IoU ($1.9163\times 10^{-3}$) are much lower than the traditional value (0.01), and they indicate that the gains should not be attributed to random error in the sample of radiographs. Collectively, these findings indicate that the dual-attention Hybrid U-Net can provide statistically significant better-quality segmentation than the no-attention baseline, which supports the role of attention mechanisms in addition to what may be explained by the EfficientNet-B3 encoder capacity.

**Figure 8:**
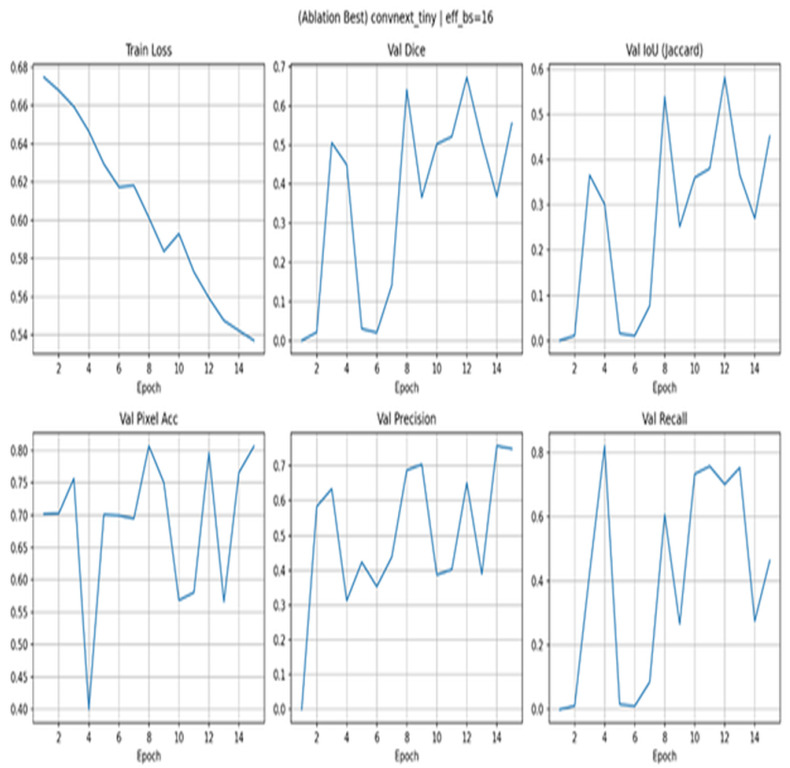
Ablation with ConvNeXt-Tiny.

Figure 8 shows the ablation with ConvNeXt-Tiny. Whereas the training loss decreases steadily (0.68–0.54), the validation curves are unstable: Dice varies between near 0 and 0.66, IoU between 0.02 and 0.56, and pixel accuracy between 0.40 and 0.80. Recall and precision vary considerably, meaning that the decision thresholds tend to be erratic and recovery of boundaries inconsistent. This configuration exhibits worse convergence and calibration than EfficientNet-B3 encoders, presumably because it does not transfer features well on limited data. These can usually help improve stability by decreasing the initial learning rate with longer warm-ups, freezing early blocks for a few epochs, and introducing weight dropout and gradient clipping, as well as reducing augmentation strength.

**Figure 9:**
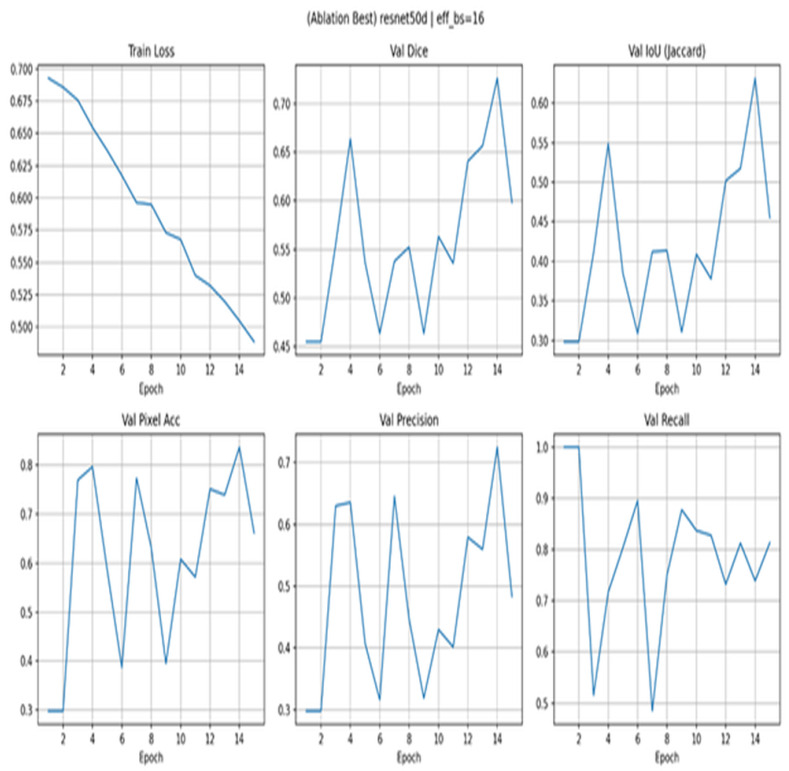
Ablation with ResNet50.

Figure 9 ablation with ResNet50 shows that despite the steadily decreasing train loss (0.69–0.49), the curves in the validation are highly fluctuating: Dice fluctuates between 0.45 and 0.72, IoU between 0.30 and 0.61, and pixel accuracy between 0.30 and 0.82. The recall spikes at the beginning, and then the oscillations appear, and precision is not regular; these are the altering decision thresholds and false positives sometimes. It is a trend that the configuration is not as well calibrated and stable as EfficientNet-B3 variants with the same recipe, most likely due to the sensitivity of the optimizer and the challenges of transferring features when working with a small scale. Regularization, augmentation, and gradient clipping are considered practical solutions, as well as less aggressive augmentation.

**Figure 10:**
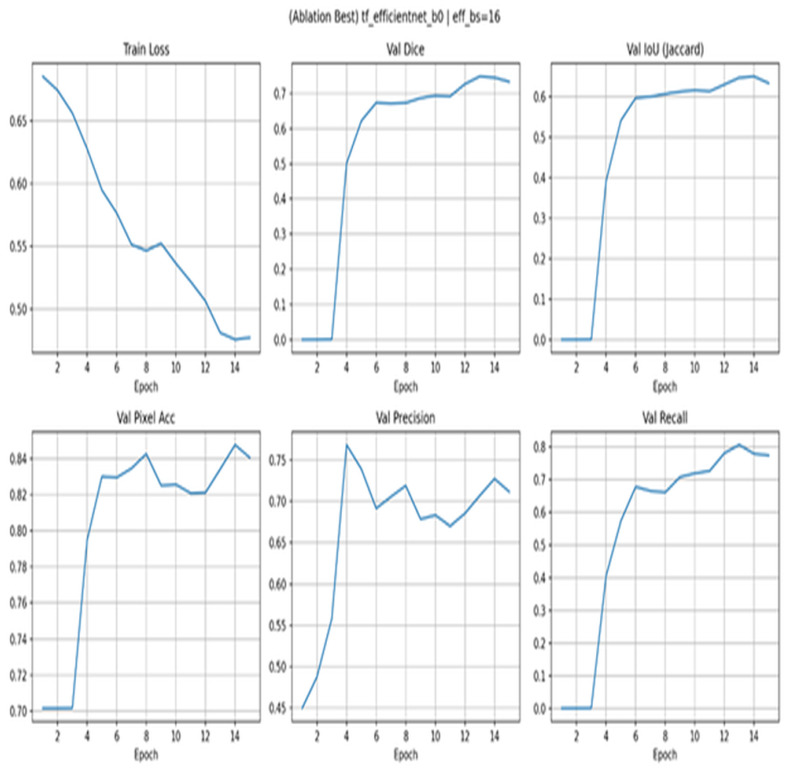
Ablation with EfficientNet-B0.

Figure 10 ablation using EfficientNet-B0 indicates Across 15 epochs, train loss decreases gradually (0.69 to 0.48) without any late overfitting signal. Validation Dice reaches the plateau phase almost instantly at 0.70–0.73 after some warming up, and IoU does the same at 0.60–0.64. The pixel accuracy is approximately 0.84–0.85 at mid-training, and it is constant. Recall monotonically increases to the highs of between 0.78 and 0.80, and precision initially peaks at about 0.75 and drops to about 0.71 towards the end, indicating weak threshold sensitivity and a few false-positive peaks.

**Figure 11:**
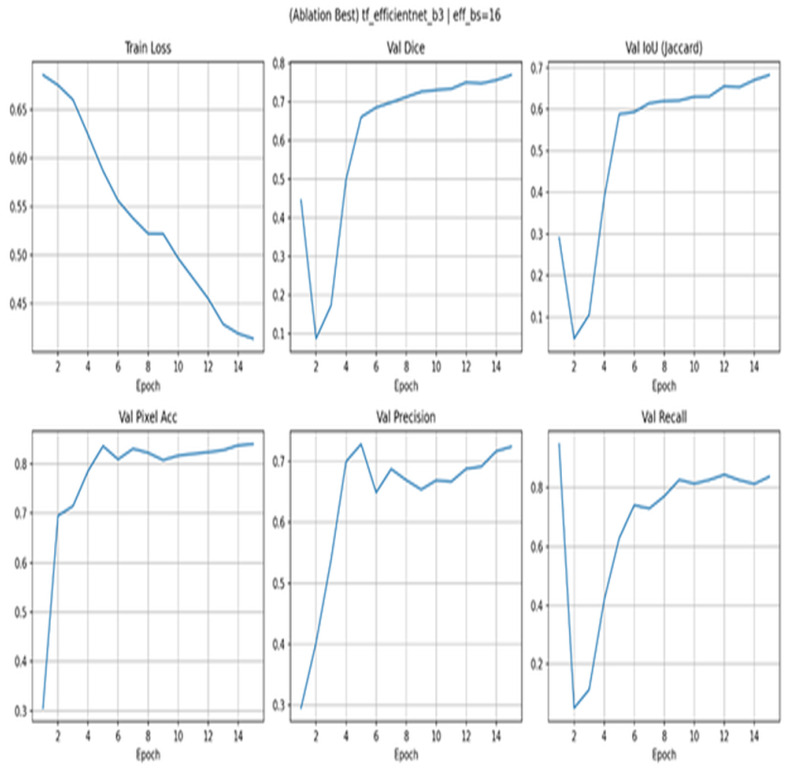
Ablation with EfficientNet-B3.

Figure 11 presents the results of the ablation using EfficientNet-B3. With the best setting of ablation, the loss of training reduces gradually to 15 epochs. Once the initial epochs have passed and validation Dice reaches similar values of around 0.78 and IoU of around 0.69, it levels off and exhibits steady convergence. Accuracy is rapidly increasing to an average of 0.84–0.85 by epoch 5 and then levels off. Precision increases more slowly, and recall is around 0.82–0.85, which is indicative of a mild recall bias. The curves do not exhibit any late-epoch degradation, indicating the lack of overfitting and proving that EfficientNet-B3 with batch size 16 is the most stable variant of the tested variants.

**Figure 12:**
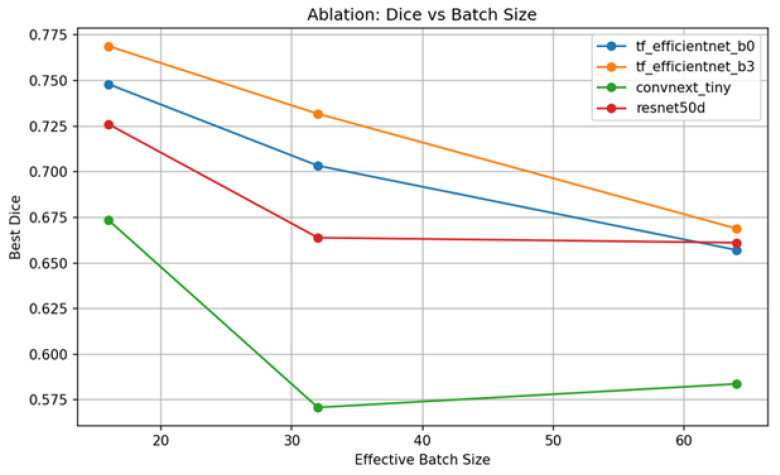
Ablation Dice vs. batch size.

Figure 12 represents ablation: Dice vs. effective batch size. The plot demonstrates the optimal validation Dice attained by every backbone with an increase in the effective batch size. Dice also decreases significantly with an increase in batch size with EfficientNet-B0 and ResNet50, respectively, indicating that small batch sizes can offer useful gradient noise/regularization. EfficientNet-B3 is again the most efficient of all sizes but also deteriorates with bigger batches. ConvNeXt-Tiny has a shallow U-shape, decreasing at the middle-sized batch and slightly increasing at the largest batch. The findings in general suggest that smaller effective batches should be used in this task and training recipe; however, in the case of large batches, learning-rate scaling or more effective regularization may be required to keep Dice.

## 4. Conclusions

This paper presented an EfficientNet-B3 encoder with a skip-aware decoder and CBAM and SCSE attention, which is a competitive, state-of-the-art radiographic segmentation model at moderate or lower computational scales. The Hybrid Attention U-Net provides Dice = 0.8426, IoU = 0.78, pixel accuracy = 0.9058, ROC-AUC = 0.9074, and PR-AUC = 0.8452 on hand X-rays and can smoothly converge and not overfit during the last epoch, as the reported tables and ablation figures support. Controlled ablation also indicates that backbone capacity is the most significant parameter in the determination of overlap quality, and EfficientNet-B3 at batch size 16 is the most stable setup in comparison to EfficientNet-B0, ResNet50, and ConvNeXt-Tiny. Quantitative gains in the form of more obvious cortical limits and reduced background leakage assist in supporting the quantitative gains, such as downstream measurement of joint-space narrowing and erosions. The combined statistical test also proves that the mechanisms of attention introduce a statistically significant difference to the no-attention level, which proves the role of the proposed Hybrid Attention U-Net is even greater than the capacity of the proposed EfficientNet-B3 encoder. Our validation is also not conducted on multiple modalities or across datasets; hence, we require strict external validation across scanners and institutions to establish how we can generalize on a real-world basis. Hand labelling can be linked to boundary noise, and no explicit label-denoising or boundary-refining was conducted after labelling. The present study is a 2D study that does not make use of volumetric context or inter-slice consistency. Finally, hyperparameters were trained using the current data and may require retraining in alternative clinical environments. These restrictions provide definite guidelines on what can be conducted in the research work in the future, such as multi-center validation, better label handling, and volumetric expansion of the proposed architecture. The major limitations of the current study is that training and evaluation are conducted on a single population X-ray dataset of the public, as well as without cross-dataset testing and independent-institutional validation. Consequently, the reported performance is to be taken as an indication of a proof-of-concept about the RF100 X-ray Rheumatology distribution, not as an indicator of totally established clinical generalizability. To partly reduce overfitting and encoder-specific bias, we employed strong data augmentation and regularization, performed controlled ablations on various encoder backbones and effective batch sizes, and shared per-image reliability statistics, rather than an overall score. These precautions, however, cannot replace actual outward validation of scanners, sites, and the population of patients. Other limitations are the use of pseudo-masks and possible boundary noise, the lack of explicit label-denoising or boundary-refinement after masking, the fact that only 2D slices were used without leveraging either volumetric or temporal context, and tuning hyperparameters that are likely to need re-tuning on other datasets. Another aspect that is still missing in our study is a direct comparison with generic segmentation models, including but not limited to nnU-Net or Canonical Attention U-Net; this will be incorporated in further research on larger, expert-labeled cohorts of RAs. Future work will involve multi-site, multi-device external validation, domain-adaptation and normalization strategies to accommodate scanner and population variations, and the expansion of the architecture to multi-class and 3D segmentation with volumetric/temporal attention, uncertainty measures, and calibration. We will also compare Hybrid Attention U-Net with nnU-Net, Attention U-Net, and other U-Nets based on EfficientNets on expert-labeled RA datasets. These measures are bound to enhance out-of-distribution generalization, reproducibility, and clinical usefulness, making the proposed model a strong foundation for further work on translation studies.

## Figures and Tables

**Table 1:** Summary of segmentation and analysis techniques in X-ray and related medical images.

S. No	Authors	Imaging Modality/ Dataset	Task Type	Techniques/ Model	Performance Metrics (as Reported)	Relation to Proposed Model
1	Wen [32]	X-ray images and 3D models	Fast X-ray simulation and bone reconstruction	Fast X-ray simulation; global optimization	MAE = 0.8082, RMSE = 0.9999	Simulation/ reconstruction, not segmentation; metrics not directly comparable.
2	Ponnusamy et al. [33]	X-ray images	Bone measurement/assessment	Deep learning with computer vision	MSE = 0.02 mm, R=0.88	Focus on measurement accuracy, not pixel-wise segmentation.
3	Brown et al. [34]	Murine micro-CT images	Bone shape modeling	Statistical shape models	Error <1% for most of the bones	Micro-CT and shape models; different modality and task.
4	Singh et al. [35]	X-ray images	Bone classification	Machine vision; ML (SVM, Naïve Bayes, k-NN, ANN)	Classification rate of 98% for SVM	Image-level classification; no dense segmentation.
5	Lo and Lai [36]	Ultrasonic images	Lesion classification	Deep learning; transformer features	Accuracy = 92%, AUC = 0.92	Different modality (ultrasound); classification task only.
6	Alarcón-Paredes et al. [37]	Thermal images; RGB photos; grip force	Multimodal classification	Artificial intelligence (multimodal fusion)	Accuracy = 94.7%	Multimodal classification; different signals and task.
7	Balaji et al. [38]	Hand radiographic images	Bone/joint classification	Convolutional neural network (CNN)	Accuracy = 97%	Same modality; image-level classification only; no pixel-wise masks.
8	Rajesh et al. [39]	Hand radiographic images	Bone classification	GLSM texture analysis	Accuracy = 88.89%	Same modality; texture-based classification; no segmentation.
9	Proposed Model	Hand radiographic images	Lesion/structure segmentation	Hybrid U-Net + EfficientNet-B3	Dice = 0.8426, IoU = 0.78, pixel accuracy = 0.9058, ROC-AUC = 0.9074, PR-AUC = 0.8452	Provides dense, pixel-wise segmentation with high overlap on the same modality.

**Table 2:** Ablation study of each backbone at 15 epochs.

Backbone	Batch Size	Dice
ConvNeXt-Tiny	16	0.67
	32	0.57
	64	0.58
ResNet50	16	0.73
	32	0.66
	64	0.66
EfficientNet-B0	16	0.75
	32	0.70
	64	0.66
EfficientNet-B3	16	0.77
	32	0.73
	64	0.67

**Table 3:** Computational efficiency and segmentation performance of encoder backbones and the proposed Hybrid Attention U-Net.

Model/Encoder	Params (M)	FLOPs (G) at Standard Input	Approx. Encoder Size (MB, FP32)	Best Dice (bs = 16) from Ablation
U-Net + ConvNeXt-Tiny encoder	28.59	4.46	114.36	0.67
U-Net + ResNet50 encoder	25.56	4.09	102.23	0.73
U-Net + EfficientNet-B0 encoder	5.30	0.39	21.20	0.75
U-Net + EfficientNet-B3 encoder	12.00	1.80	48.00	0.77

**Table 4:** Ablation of attention modules on the EfficientNet-B3 U-Net (fixed bs = 16, 15 epochs).

Attention Setting (Decoder)	Dice	IoU	Boundary F1	Leakage Rate
No attention	0.7921	0.7176	0.6409	0.2263
CBAM only	0.7606	0.6682	0.5187	0.2812
SCSE only	0.8016	0.7274	0.6255	0.2232
CBAM + SCSE	0.7741	0.6846	0.5491	0.2820

**Table 5:** Reliability of segmentation metrics on the held-out set (per-image).

Metric	Mean ± SD	95% CI
Dice	0.7872±0.3012	(0.6813,0.8815)
IoU	0.7212±0.3020	(0.6151,0.8166)
Pixel accuracy	0.9058±0.2320	(0.7851,0.9388)
Boundary F1	0.7231±0.2318	(0.6426,0.7968)

**Table 6:** Paired statistical test between baseline (no attention) and proposed dual-attention model.

Metric	Baseline Mean	Proposed Mean	*p*-Value
Dice	0.7323	0.7780	$4.8702\times 10^{-3}$
IoU	0.6485	0.7217	$1.9163\times 10^{-3}$

## Data Availability

The dataset and the code are available in the link https://universe.roboflow.com/roboflow-100/x-ray-rheumatology (accessed on 30 October 2025).
